# Comprehensive transcriptomic analysis indicates brain regional specific alterations in type 2 diabetes

**DOI:** 10.18632/aging.102196

**Published:** 2019-08-26

**Authors:** Zhe Zhou, Yizhang Zhu, Yang Liu, Yuxin Yin

**Affiliations:** 1 Institute of Systems Biomedicine, School of Basic Medical Sciences, Peking University Health Science Center, Beijing 100191, China; 2 Department of Pathology, School of Basic Medical Sciences, Peking University Health Science Center, Beijing 100191, China

**Keywords:** brain, transcriptome, type 2 diabetes, co-expression network, caudate

## Abstract

Type 2 diabetes (T2D) can result in a number of comorbidities involving various organs including heart, eye, kidney and even the brain. However, little is known about the molecular basis of T2D associated brain disorders. In this study, we performed a comprehensive transcriptomic analysis in a total of 304 T2D samples and 608 matched control samples from thirteen distinct brain regions. We observed prominent difference among transcriptomic profiles of diverse brain regions in T2D. The most striking change was found in caudate with thousands of T2D-associated genes identified, followed by hippocampus, while nearly no transcriptomic change was observed in other brain regions. Functional analysis of T2D-associated genes revealed impaired synaptic functions and an association with neurodegenerative diseases. Co-expression analysis of caudate transcriptomic profiles unveiled a core regional specific module that was disorganized in T2D. Sub-modules consisting of regional markers were enriched in T2D risk single nucleotide polymorphisms (SNPs) and implied a causal link with T2D. Hub genes of this module include *NSF* and *ADD2*, the former of which has been associated with T2D and neurogenerative diseases. Thus, our work provides useful information for further studies in T2D associated brain disorders.

## INTRODUCTION

Type 2 diabetes (T2D), which is defined as a group of metabolic disorders characterized by both insufficient insulin secretion and insulin resistance, is a major subtype of diabetes and accounts for more than 90% of it [1]. Globally, an estimated 422 million adults are living with diabetes and over 1.5 million deaths are caused by it every year [2]. T2D is a complex disease with numerous systemic complications including heart attack, kidney failure, vision loss and peripheral nerve damage [3]. Specific cell types and molecular pathways involved in majority of these complications have been well discussed [4]. Nonetheless, scant attention has been paid to the influence of T2D on the structure and function of the central nervous system (CNS), which is the most important system in the body.

Recent evidence suggests that T2D doubles the risk of vascular dementia and neurodegenerative diseases in older age [5–8]. Longitudinal cohort studies have also linked T2D with significant decline in processing speed [9, 10], executive function [9, 11, 12], memory [9–11] and verbal fluency [10]. Imaging studies in diabetic brains reported global brain atrophy [13] and microstructural lesions in the cerebral gray and white matter [14]. Given the above observations, it is of great need to understand the mechanism of these disorders and to identify molecular targets and pathways involved. Several studies reported the potential role of inflammation, defective insulin signaling and mitochondrial dysfunction in T2D-associated CNS disorders [15–17]. Nonetheless, few of them were performed on human brains with sufficient samples. Moreover, human brain is composed of diverse regions which execute different functions, whilst majority of these studies focused on specific brain regions. To the best of our knowledge, the difference between diverse brain regions in T2D remains elusive.

Disruption in the normal gene expression profile of various tissues is an important link between T2D and its complications [18]. With the advent of high-throughput sequencing, it is feasible to study the brain transcriptomic changes associated with T2D in various brain regions. The Genotype-Tissue Expression (GTEx) project [19] provides whole transcriptomic profiles of 13 brain regions derived from T2D subjects and healthy controls, thus making it one of the most comprehensive datasets for studying region-specific T2D-asssociated transcriptomic changes.

In the present study, we first performed differential expression analysis in 13 brain regions samples and identified regional specific T2D-associated genes (DAGs). We found that majority of the brain was immune to T2D, while great transcriptomic changes were observed in caudate and hippocampus. Next, we explored the distributions and functions of these regional specific DAGs. Rather than analyzed at individual gene level, we also performed co-expression analysis on T2D-involved brain regions. Core modules and hub genes which might help unravel the underlying mechanisms were identified by systemic analysis. Hereby, our analysis provides a basis for further researches of T2D-associated brain alterations.

## RESULTS

### Identification of DAGs in 13 brain regions

**Figure 1:**
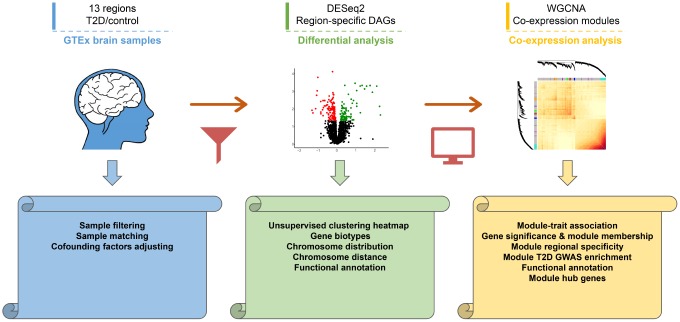
**Workflow diagram of the study design.** First, transcriptomic profiles of 13 human brain regions were derived from GTEx dataset. Second, differential analysis was performed to investigate regional specific changes. Distributions and functional annotations of DAGs were analyzed subsequently. Finally, Co-expression analysis was performed in caudate and hippocampus to study core modules and hub genes. The volcano plot and heatmap were generated using random sampling data of caudate transcriptome.

The major workflow of present study was shown in Figure 1. Control samples were matched with T2D cases to avoid bias resulting from cofounding factors. A total of 304 T2D samples and 608 matched control samples from thirteen distinct brain regions were then obtained. No significant differences were detected between matched samples for most of the covariates (Supplementary Figure 1 and Supplementary Table 1). Sample distribution after matching was plotted for each brain region, as shown in Supplementary Figure 2.

**Figure 2:**
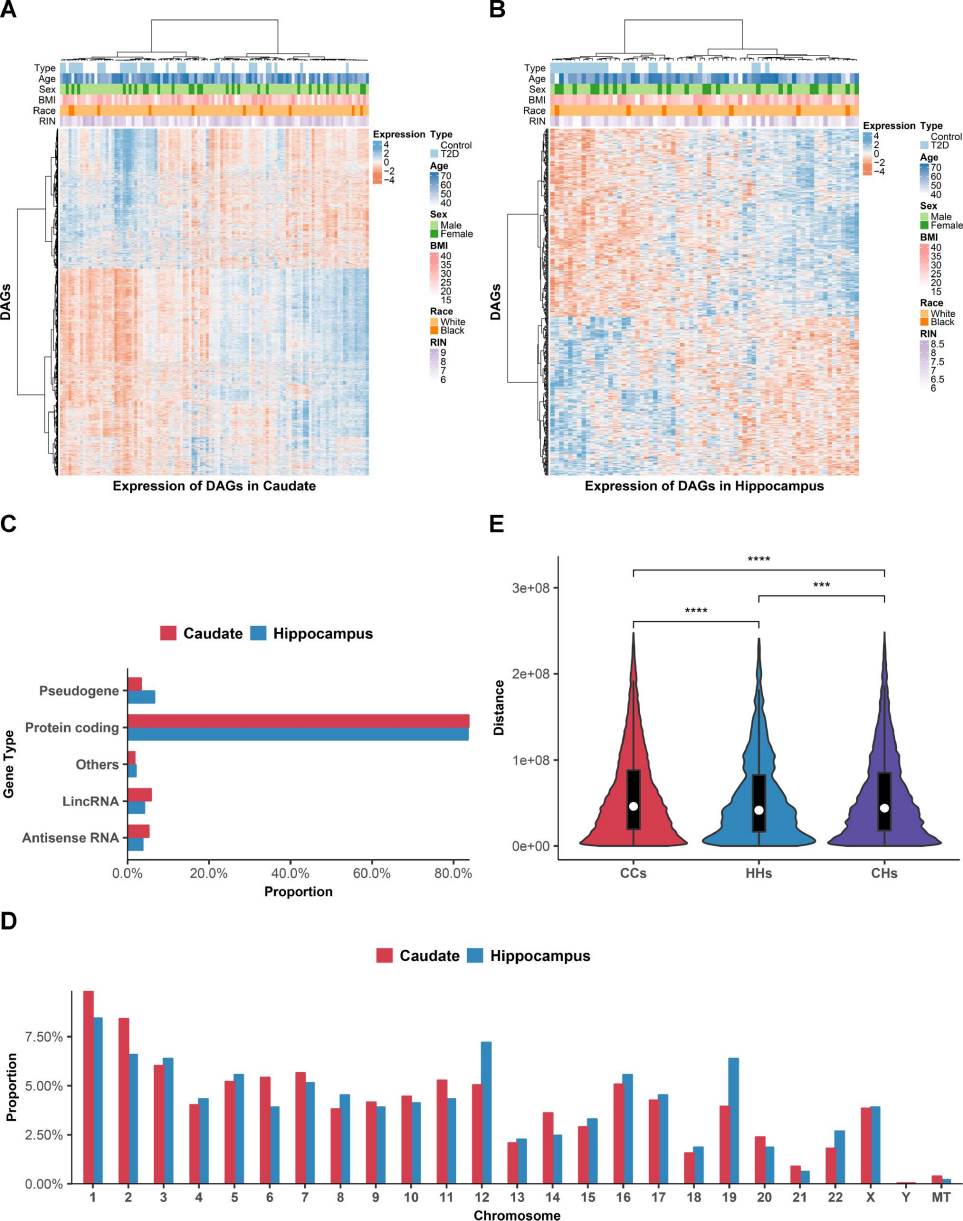
**Distributions of DAGs.** (**A** and **B**) Heat maps of DAGs (row) on samples (column) were shown for caudate (**A**) and hippocampus (**B**). (**C**) Gene biotype categories of DAGs according to GENCODE. X-axis shows the proportion to the total DAGs in each brain region. (**D**) Chromosomal distribution of DAGs. Y-axis shows the proportion to the total DAGs in each brain region. (**E**) The violin plot comparing the intra-chromosomal distances between DAGs in different brain regions. ^***^P < 0.001; ^****^P < 0.0001, Wilcoxon’s test. CCs, distance of DAGs within caudate group; HHs, distance of DAGs within hippocampus group; CHs, distance of DAGs between caudate and hippocampus group.

To identify DAGs, we performed generalized linear regression for genes against T2D status in each brain region using DESeq2 [20], with known and surrogate variables adjusted. At a 5% false discovery rate (FDR), less than 10 or even no DAGs were identified in most regions, indicating that T2D had no notable effect on transcriptome of the majority of human brain. Nevertheless, regulation of a considerable number of genes were disturbed in two regions, namely caudate (2939 DAGs) and hippocampus (486 DAGs) (Table 1). Among all the DAGs, only 178 genes were dysregulated in more than one tissue, and expression of all the ‘multi-hit’ DAGs responded to T2D status in the same direction except for 3 of them. This result indicated that different brain regions might associate with T2D in a region-specific manner. The details of DAGs in each brain region are provided in Supplementary Table 2. Control cases and T2D samples can be distinguished roughly in unsupervised clustering heatmap (P-value: 2.19eE-03 for caudate and 7.78E-07 for hippocampus, Fisher’s exact test) using identified DAGs (Figure 2A and 2B).

**Table 1:** Numbers of DAGs in 13 brain regions.

**Regions**	**FDR < 10%**			**FDR < 5%**			**FDR < 1%**		
	**total**	**up**	**down**	**total**	**up**	**down**	**total**	**up**	**down**
Amygdala	59	7	52	6	0	6	1	0	1
Anterior Cingulate Cortex	3	0	3	1	0	1	1	0	1
Caudate	4548	1910	2638	2939	1186	1753	1049	448	601
Cerebellar Hemisphere	12	4	8	2	0	2	0	0	0
Cerebellum	1	0	1	0	0	0	0	0	0
Cortex	0	0	0	0	0	0	0	0	0
Frontal Cortex	0	0	0	0	0	0	0	0	0
Hippocampus	1143	527	616	486	223	263	82	31	51
Hypothalamus	1	0	1	0	0	0	0	0	0
Nucleus Accumbens	66	26	40	0	0	0	0	0	0
Putamen	0	0	0	0	0	0	0	0	0
Spinal Cord	3	1	2	1	0	1	0	0	0
Substantia Nigra	0	0	0	0	0	0	0	0	0

Note: Columns “total”, “up” and “down” list the number of total, up- and down-regulated DAGs respectively. Results derived from using three different FDR cutoffs (10%, 5% and 1%) are shown.

In view of the fact that caudate had the maximum sample size among all the 13 brain regions and the marked influence of sample size on differential analysis, we cannot evaluate the impact of T2D on diverse brain regions according to the number of DAGs directly. Therefore, we randomly selected samples with size ranging from 10 T2D samples versus 20 matched controls (the minimal size in 13 brain regions) to maximal number of each brain regions, with five T2D samples and ten matched controls added each time, and then bootstrapped 100 times. As expected, caudate got the largest number of DAGs at a 5% FDR level, followed by hippocampus, regardless of the sample size (Supplementary Figure 3). This result indicated that diverse brain regions were altered differentially in T2D. Furthermore, caudate and hippocampus might be the brain regions with the most significant transcriptomic changes in T2D.

### Distributions and functional annotations of DAGs in various brain regions

To study the distributions of DAGs in various brain regions, we first categorized the DAGs according to gene biotypes in GENCODE [21]. Protein coding genes account for majority of DAGs (83.9% of caudate DAGs and 83.5% of hippocampus DAGs, Figure 2C). Whilst there was also a small subset of DAGs comprised of non-coding genes, including antisense RNA, pseudogene, lincRNA and others, implying the function of these non-coding genes in pathological process of diabetic brain.

Next, we investigated the chromosomal distribution patterns of DAGs, which showed that they were widespread across chromosomes (Figure 2D). More than 95% of DAGs located on autosomes and the overall distribution of caudate and hippocampus DAGs were similar. However, the proportion of DAGs located on small, gene-rich chromosomes (chr16-22) was slightly higher in hippocampus than in caudate.

As genes involved with the same disease process tend to locate adjacently [22], we then calculated the genomic distance of each pair of DAGs on the same chromosome (Figure 2E). Results showed that distances of DAGs within hippocampus group were the smallest compared with distances of DAGs within caudate group (P-value=8.95E-14) and between groups (P-value=1.11E-4). Surprisingly, distances of DAGs between groups were also smaller than distances of DAGs within caudate group (P-value = 1.82E-27). In general, above results suggested regional specific distribution of DAGs in various brain regions.

To obtain a functional overview of DAGs in different brain regions, we dissected functional annotations of the up- and down-regulated DAGs respectively. As a result, top Gene Ontology (GO) biological process terms enriched with down-regulated DAGs in caudate and hippocampus both related to synaptic functions (Supplementary Table 3), which have been reported to impaired in diabetic brain [23]. Nonetheless, functions of up-regulated DAGs tend to be more regional specific. In caudate, up-regulated DAGs enriched in terms like cilium movement and cilium organization, implying the hidden association of T2D and ciliopathy [24]. However, for up-regulated DAGs in hippocampus, terms related to muscle function and muscle tissue morphogenesis were enriched. Kyoto Encyclopedia of Genes and Genomes (KEGG) pathway enrichment analysis revealed that DAGs in caudate also enriched in neurodegenerative diseases pathways such as Alzheimer disease, Huntington’s disease and Parkinson’s disease. In consideration of aforementioned association between T2D and neurodegenerative diseases [6–8], the caudate nucleus and related DAGs might play a vital role in these processes.

### Co-expression analysis of caudate and hippocampus

Although numerous DAGs were identified in caudate and hippocampus, limited information could be provided by individual genes. Moreover, DAGs determined by differential expression analysis methods were biased to genes with large change in expression, while overlooking the coordination of gene expression. To integrate the expression difference into a systems-level context, we performed weighted gene co-expression network analysis (WGCNA) on caudate and hippocampus. We detected 14 modules in caudate (Supplementary Figure 4 and Supplementary Table 4) and 40 modules in hippocampus (Supplementary Figure 5 and Supplementary Table 5). The average size of caudate modules was larger than hippocampus (P-value: 9.43E-03, Wilcoxon test), which implied a stronger co-expression of transcriptomic profiles in caudate.

**Figure 3:**
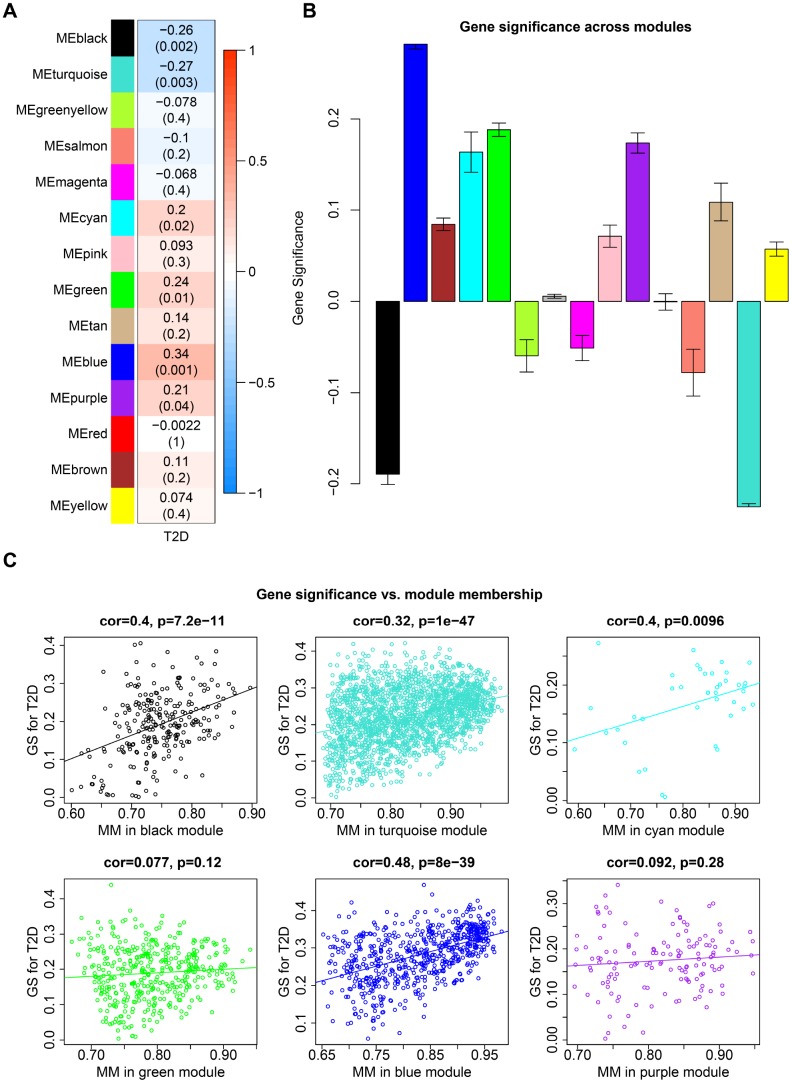
**Co-expression modules in caudate.** (**A**) Each row corresponds to a module eigengene and column indicate T2D status. The table were colored by correlation according to the legend. Each cell contains the corresponding correlation and P-value. (**B**) Each bar indicates the average of gene significance measure for all genes in a given module. (**C**) GS vs. MM plot for modules significantly correlated with T2D status. Each point corresponds to an individual gene within a given module, which was plotted by GS on the y-axis and MM on the x-axis. The regression line, correlation value and P-value were shown for each module.

Among the 14 co-expression modules in caudate, eigengenes of 4 modules (cyan, green, blue and purple) were positively correlated (up-regulated) with T2D, while black and turquoise module were negatively correlated (down-regulated) with T2D (Figure 3A, P-value < 0.05, Student t test). No modules correlated with any of the potential confounding variables which have been regressed out. Gene significance (GS) varied dramatically across modules, which also indicated the different levels of correlation between co-expression modules and T2D (Figure 3B). To examine gene constitution of particular modules, we plotted GS against module membership (MM) for T2D-associated modules (Figure 3C). Significant correlations were detected in black, turquoise and blue modules, illustrating that genes significantly associated with T2D status are often also the important elements of these modules. This result confirmed the crucial role of these modules in type 2 diabetes process.

In hippocampus, five of the 40 co-expression modules positively associated with T2D and another five modules negatively associated (Supplementary Figure 6A, P-value < 0.05, Student t test). The mean GS values across modules were shown in Supplementary Figure 6C. Among the 10 T2D-associated modules, significant correlations of GS and MM were detected in 7 modules (Supplementary Figure 6B).

### Cell-type and regional specificity of co-expression modules

**Figure 4:**
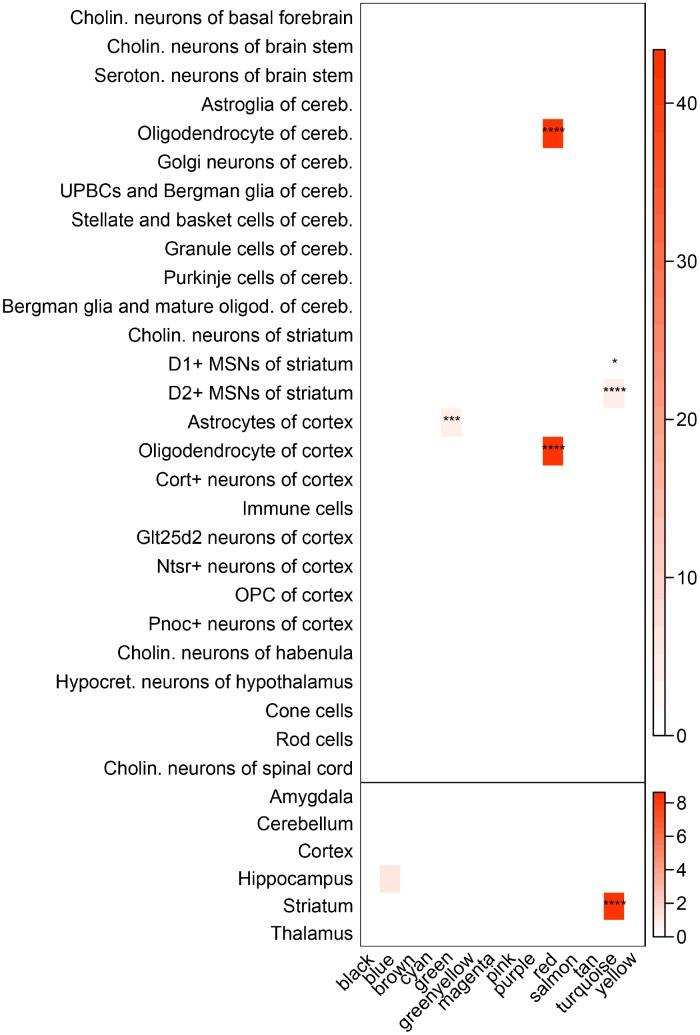
**Enrichment of brain regional and cell-type markers in caudate modules.** (Top) Enrichment of caudate modules in markers of various neuronal and glial cell types. (Bottom) Same as above, but using markers for different brain regions. Asterisks indicate significant enrichment after Bonferroni adjustment. ^*^P < 0.05; ^**^P < 0.01; ^***^P < 0.001; ^****^P < 0.0001, Fisher’s Exact test. Cholin., cholinergic; seroton., serotonergic; cereb., cerebellum; UPBCs, unipolar brush cells; oligod., oligodendrocyte; MSNs, medium spiny neurons; hypocret., hypocretinergic.

Disorder of regional specific gene networks usually results in impairment of regional specific functions. Hence, it is of great value to determine whether brain regional specific modules were disturbed in T2D, which might help unravel the underlying mechanisms of T2D-associated brain disorders. By testing the enrichment of distinct regional and cell-type specific expression markers [25], several modules in caudate were found to be significant at pSI 0.0001 (Figure 4). For instance, the green module, positively correlated with T2D, was significantly enriched in cortical astrocytes markers. The red module was significantly enriched in expression markers of oligodendrocyte of cortex and cerebellum, whilst eigengene of this module did not show prominent correlation with the T2D status. The most surprising result is the turquoise module, which was found to be striatum specific at every pSI (Supplementary Table 6). Cell-type markers of two major neuronal populations in striatum: Drd1-expressing and Drd2-expressing medium spiny neurons (D1- and D2-MSNs) were also enriched in turquoise module at every pSI. In consideration of the transcriptional association between turquoise module and T2D, this module could be determined as a striatum-specific module which was disturbed in T2D status.

However, in hippocampus, there were no T2D-associated modules enriched in any regional or cell-type markers at pSI 0.0001 (Supplementary Figure 7 and Supplementary Table 7). The darkolivegreen module was the only module enriched in hippocampus markers. Nevertheless, eigengene of this module had a weak correlation with T2D. Therefore, the darkolivegreen module may not account for the alteration of hippocampus in T2D. Next, we focused only on caudate to further study the correlation between T2D and these putative core modules.

### Identification of modules genetically associated with T2D in caudate

Though genes in modules specific to caudate were dysregulated in T2D, it was unclear whether these impairments were only consequences of T2D or genetically associated with T2D. To identify the causal link of modules to T2D, we tested for their enrichment in T2D and height (as a negative control) associated single nucleotide polymorphisms (SNPs) from large genome-wide association study (GWAS) data sets.

**Figure 5:**
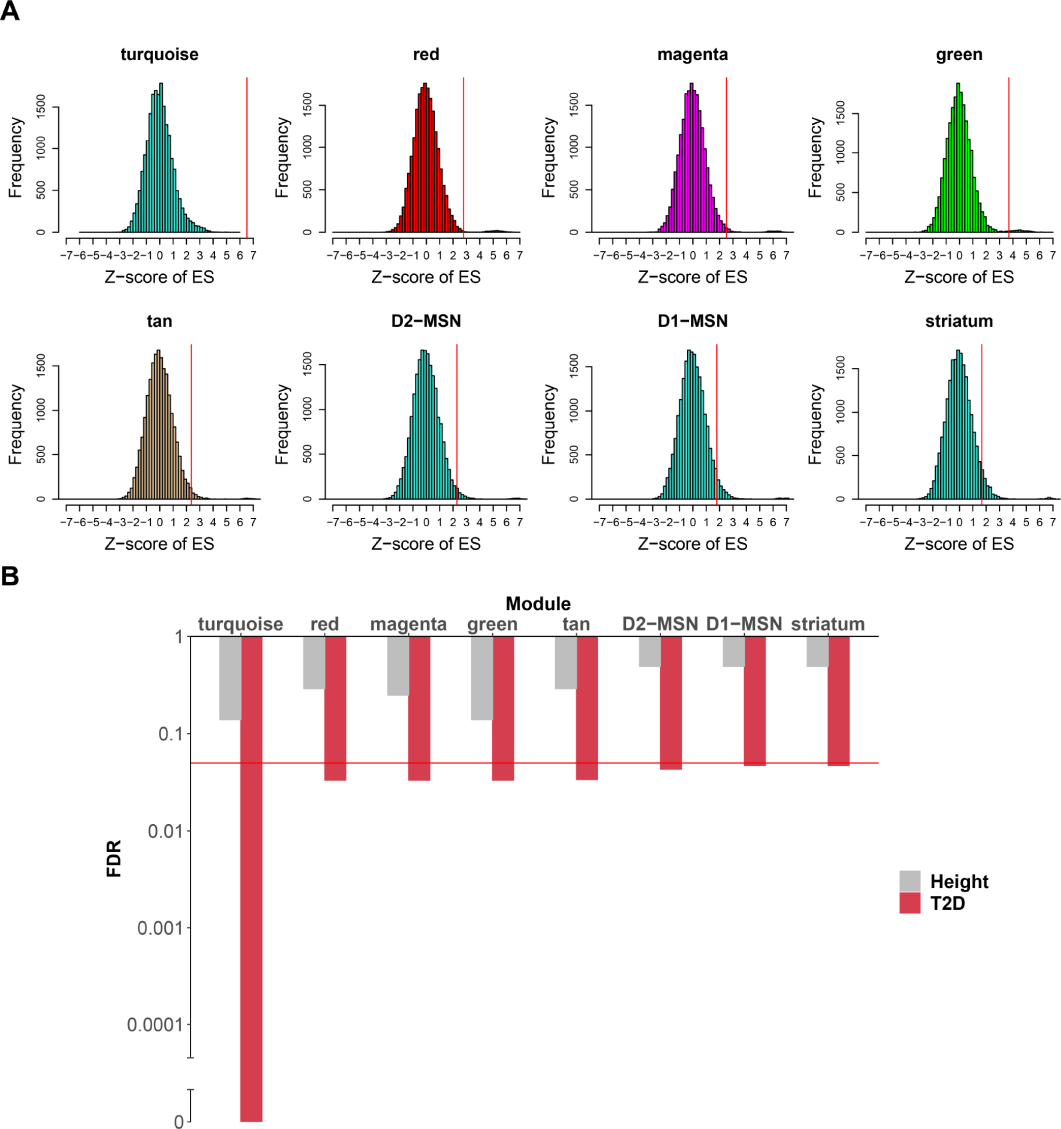
**Caudate modules enriched in T2D genetic signals.** (**A**) For each module (or sub-module), the null distribution of T2D SNPs enrichment scores generated by 20,000 random permutations is shown. Real enrichment scores were depicted by red vertical lines. Modules were considered significant if FDR < 0.05. (**B**) For each module in (**A**), enrichment FDR for T2D SNPs are shown by histogram compared to height SNPs. Y-axis was log10 transformed and broken axis was used to show zero value. The red horizontal line marks the FDR threshold for significance, which is 0.05. FDR, false discovery rate.

In caudate, the turquoise module exhibited highly significant enrichment in T2D risk SNPs, while no such significant result was found in height-associated SNPs (Figure 5A, 5B and Supplementary Table 8). As the turquoise module was also highly enriched in striatum and MSNs markers, it was of great interest whether the T2D risk SNPs signal of this module was in connection with these specific markers. To explore this, we extract sub-modules of the turquoise module which only contain striatum, D1-MSNs or D2-MSNs specific markers at pSI 0.01 (sub-module derived at pSI 0.001 and 0.0001 was too small), respectively, and then performed the same enrichment method. All the three sub-modules were significantly enriched in T2D risk SNPs compared with height, especially for D2-MSNs. It can be inferred that the genetic risk for T2D associated with the turquoise module might distribute mainly across striatum and MSNs specific genes. Another 4 modules (red, magenta, green and tan module) were also relatively significant compared to negative control. However, only eigengene of green module was correlated with T2D and this module was enriched in cell-type markers of astrocytes of cortex.

### Functional annotations and hub genes of caudate modules

**Figure 6:**
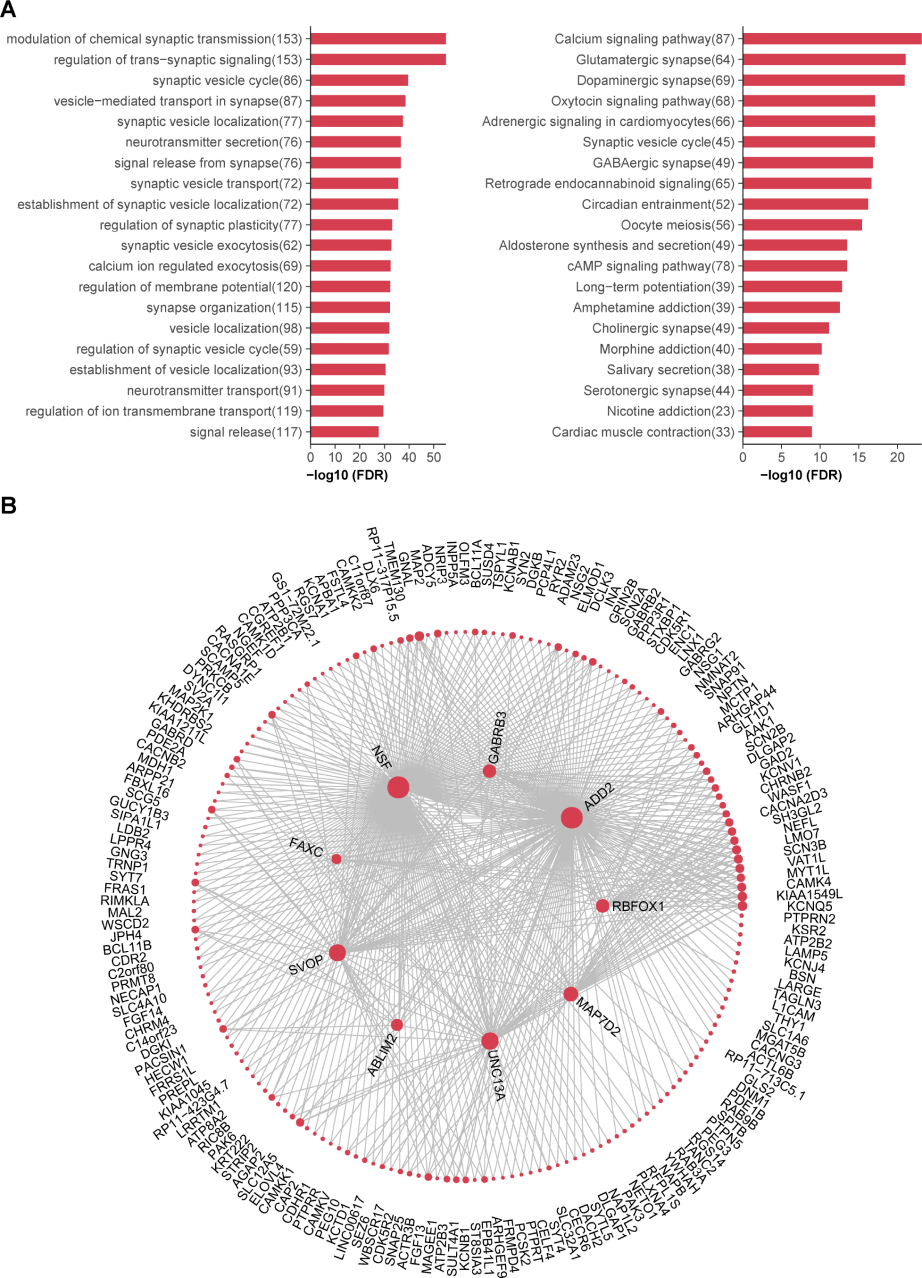
**Functional annotations and hub genes of caudate modules.** (**A**) (Left) Top 20 GO biological processes significantly enriched in turquoise module. (Right) Top 20 KEGG pathways significantly enriched in turquoise module. Numbers in the parenthesis indicate the numbers of genes associated with the respective terms. (**B**) Network plots showing top 500 connections in turquoise module; genes with most connections (hub genes) are shown in center. The size of each dot is proportional to log2 (number of connections for each gene).

Previous studies have shown that co-expressed genes tend to be functional related [26], we therefore studied the functional annotations of interested modules. Actually, although more than half of input genes were unassigned to specific co-expression modules, there were still large overlaps between DAGs and several modules. For instance, the blue module accounted for nearly 40% of up-regulated DAGs in caudate and a half of down-regulated DAGs were assigned to turquoise module (Supplementary Table 9). Nonetheless, the co-expression modules also harbored non-T2D-associated genes and had a greater power to delineate T2D-relevant transcriptional changes compared with DAGs. As an illustration, similar but more significant pathways were enriched for the blue module than up-regulated DAGs (Supplementary Table 10). The turquoise was also enriched in terms related to synaptic functions more significantly (Figure 6A), compared with down-regulated DAGs. In terms of other aforementioned T2D-associated modules, the cyan module associated with ribosome and the purple module is an immune module, revealing corresponding dysfunction in T2D brain. It was worth mentioning that the green module enriched in many metabolic processes and axonogenesis, in accordance with its enrichment in cortical astrocytes marker.

Other than taking the turquoise module as a whole, we further investigated enriched pathways of striatum and MSNs sub-modules. All of these were enriched in pathways related to learning, memory and cognition (Supplementary Table 10). Taken together, the turquoise module was considered as a core functional module of caudate and its related synaptic impairments may contribute to the cognition decline in T2D.

Finally, we tried to identify highly connected hub genes for the turquoise module (Figure 6B). The top two hub genes were *NSF* (N-ethylmaleimide-sensitive factor, Entrez ID 4905) and *ADD2* (adducin 2, Entrez ID 119), both of which were highly expressed in brain. NSF is essential for neurotransmitter release, together with other key factors of synaptic fusion machinery, such as SNAREs (soluble NSF attachment protein receptors) and SNAP (soluble NSF adaptor protein). They have been reported to play an important role in both diabetes and neurodegenerative disorders [27]. *ADD2* encodes the beta subunit of adducins, which are a family of cytoskeletal proteins. Polymorphism of *ADD1* gene (encoding the alpha subunit, Entrez ID 118) has been reported to associated with T2D [28], while little is known about the relationship between *ADD2* and T2D. Thus, further studies are needed to explore the roles of these hub genes in T2D brain.

## DISCUSSION

Identification of genes and pathways altered in diabetic brains may provide insights into the mechanism and prognosis of T2D-associated CNS disorders. In the present study, we delineated transcriptomic changes of 13 brain regions in T2D and observed prominent difference among diverse brain regions. Although transcriptome of majority of the human brain was stable in T2D, a considerable number of DAGs were identified in caudate and hippocampus. Even anatomically adjacent regions might also show dramatic difference in T2D. A notable example was caudate and putamen, the former of which has the most abundant number of DAGs, while none are identified in the latter. Functional annotations indicate that the down-regulated DAGs in caudate and hippocampus are both enriched in synaptic pathways, whilst the up-regulated DAGs have regional specific functions.

We also performed co-expression analysis on these two regions and observed different co-expression patterns. We identified a turquoise module that harbors a half of down-regulated DAGs in caudate while performed better in delineating transcriptomic changes of caudate. This module is enriched in regional markers of striatum and cell-type markers of its two major neuronal populations, consistent with previous evidence that GABAergic neurons in striatum is negatively affected in T2D rats [29]. Of particular interest, the turquoise module is enriched in T2D risk SNPs, implying their potential role in etiology of diabetes. Dissection of functions of turquoise modules and its sub-modules has revealed their core role in synaptic transmission and cognition. Moreover, the identified hub genes of the turquoise modules might play a vital role in coordination of involved genes. For instance, the top hub gene *NSF* can link T2D with neurodegenerative diseases together with SNAREs. It is of note that although no T2D-associated CNS complications were observed in GTEx donors, remarkable alterations have been existed in diabetic brain. Hence, early interventions to prevent diabetes-related CNS complication were recommended. There is no such global gene expression profiling of multiple brain regions in T2D has been reported. As type 2 diabetes and CNS disorders getting prevailing, our study provides a broader horizon for further research.

Given the association between T2D and its related brain alterations, much attention has been directed to the hippocampus and cognitive decline [30]. However, pooled analysis showed that the hippocampus was not more severely affected than the rest of the brain [13]. In our study, large transcriptomic changes of hippocampus were observed, whilst the changes seemed weaker than caudate and we were not able to identify convergent core modules for it. Further researches were still required for better understanding the role of hippocampus in T2D related brain alterations.

On the other hand, the caudate has also been associated with cognitive impairment [31, 32]. The caudate and putamen together constitute the striatum, which is a part of basal ganglia. Various nuclei of basal ganglia are functionally delineated along corticostriatal lines. The caudate is associated with selection of appropriate sub-goals based on an evaluation of action-outcomes, whereas its nearest neighbor, the putamen, appears to be involved in simpler motor control [33]. This might help explain the striking difference in transcriptomic changes of caudate and putamen in T2D. The cognitive function of caudate, along with caudate abnormality observed in T2D [34, 35], also provides support for the potential role of caudate in T2D associated brain alterations.

Nevertheless, there are also limitations of the present study. First, due to the rarity of T2D postmortem brain samples, the sample size of GTEx database is still relatively small. Further investigation with larger sample size is recommended to reduce noise and draw more reliable conclusions. Second, gene expression is a complex trait influenced by various factors. Although we have tried our best to match samples as well as control known and hidden factors in our pipeline, bias might still exist. Sample pairing in the experiment design stage can further reduce possible bias. Third, there are no quantitative indicators of hyperglycemia, such as glycated hemoglobin (HbA1c), in GTEx dataset. Hence, transcriptomic changes associated with blood glucose level could not be determined, which may provide useful information for explanation of etiology of T2D complications. Ultimately, more comprehensive studies are expected in the future to deepen our understanding on this topic.

## MATERIALS AND METHODS

### GTEx tissues and subjects

The GTEx project (v7, released in June 2017) provides expression data of 13 human brain regions from 752 post-mortem donors. Two of the brain regions were initially sampled from cerebellum and cortex preserved using the PAXgene tissue preservation system, and another 11 regions were subsequently sampled from frozen brains, including amygdala, anterior cingulate cortex, caudate, cerebellar hemisphere, frontal cortex, hippocampus, hypothalamus, nucleus accumbens, putamen, spinal cord and substantia nigra. Details regarding the sample collection, RNA sequencing and data processing are available at GTEx consortium paper [36].

To draw reliable conclusion, we only keep high-quality sequencing samples with RINs > 6.0. Cases with type 1 diabetes or unknown T2D status, and races other than black or white were excluded from this study.

Confounding factors including age [37], gender [38], race, BMI [39] and RIN [40] have been reported to be correlated with gene expression. To avoid bias, we used an optimal matching algorithm in R package MatchIt [41] to balance them between control and T2D groups, with optimal ratio of 2:1. The number of remaining matched samples of 13 brain regions ranged from 30 to 108.

### Identification of regional specific DAGs

DESeq2 [20] (v1.22.2) was employed on all of the 13 brain regions to identify regional specific DAGs using raw read counts. Independent filtering of genes with low read counts was performed automatically by DESeq2 with alpha=0.05, and genes remained were referred to as ‘detectable genes’. To correct the known covariates as well as remove inferred hidden confounders in GTEx expression data, we employed “svaseq” function in sva R package [42] to identify 3 surrogate variables. The known confounding factors and surrogate variables were then added to the DESeq2 design, and negative binomial (NB) generalized linear regression model (GLM) was performed. Benjamini-Hochberg (BH) algorithm was used to adjust the Wald test P-values for multiple testing. Raw counts data were transformed to continuous, homoscedastic regularized logarithm transformed (r-log) values for further analysis.

### Genomic distance and functional annotations

Genomic distance of each pair of DAGs on the same chromosome was calculated according to their genome coordinate positions. Wilcoxon rank-sum test was used to compare the distributions of intra- and inter-groups.

Functional enrichment analysis was performed using R package clusterProfiler [43] to identify significant GO biological process terms and KEGG terms. The FDR adjustment for P-value was made using Benjamin-Hochberg procedure and an FDR cutoff of 0.05 was used.

### Weighted gene co-expression network analysis

To regress out uninterested sources of large variation, linear models containing age, gender, race, BMI, RIN and surrogate variables were fitted on the r-log data for all of the ‘detectable genes’ in each brain region, respectively. Then co-expression analysis was performed on residuals using WGCNA R package [44]. The soft-thresholding power were picked according to the scale-free topology criterion, and a singed gene network was constructed using blockwiseModules function in a single block with parameters mergeCutHeight = 0.15, minModSize = 40 and minKMEtostay = 0.7.

The module eigengene (ME) is defined as the first principal component of a given module, which can be considered as a representative of the gene expression profiles in a module. Module membership (MM) is a measure of gene-to-module membership by correlating its gene expression profile with the module eigengene of a given module. Gene significance (GS) was defined as the correlation between the gene and the T2D status.

Module graphs showing the top 500 connections were plotted using the iGraph [45] R package. Hub genes were defined as top 5% in number of connections.

### Cell-type and regional specificity analysis

R package pSI [46], which provides lists of expression markers for diverse brain regions and cell types, was used to perform Fisher’s Exact test for regional or cell-type marker enrichment in co-expression modules at different specificity index thresholds (pSI 0.01, 0.001 and 0.0001; pSI 0.05 was deprecated for increased false positives). Regional markers were derived from Atlas of the developing Human Brain (www.brainspan.org), while cell-type markers were originally identified using translational profiling of genetically tagged cell lines purified from mouse brain [25].

### GWAS set enrichment analysis

Enrichment for GWAS signal was conducted as previously described [47]. SNPs and their associated P-values were from large GWAS data sets of T2D [48] and height [49]. SNPs were assigned to genes if located within gene boundaries with additional 20kb on 5’ end and 10kb on 3’ end. The most significant SNP of each gene was selected, and then all of the genes were ranked with associated scores (-log_10_ P-value) to calculate gene set enrichment score (ES) based on the Kolmogorov-Smirnov running-sum statistic using GSEA-P [50]. The null distribution of ES was generated by 20,000 random permutations of gene labels and associated scores. Enrichment scores were scaled by subtracting the mean and dividing by the standard deviation of permutation scores to correct for the gene set size, and empirical P-values were determined by the resulting z-scores.

### Data and resource availability

The GTEx gene expression data and phenotype data were downloaded from dbGaP (http://www.ncbi.nlm.nih.gov/gap) under accession number phs000424.v7.p2. Statistical analysis was performed on R (v3.5.0). Scripts are available at https://github.com/ZedekiahZhou/T2D_Brain. All data generated or analyzed during this study are included in the published article (and its online supplementary files).

## Supplementary Material

Supplementary Figures

Supplementary Table 1

Supplementary Table 2

Supplementary Table 3

Supplementary Table 4

Supplementary Table 5

Supplementary Table 6

Supplementary Table 7

Supplementary Tables 8 and 9

Supplementary Table 10
