# Supplementary material for: Comprehensive transcriptomic analysis indicates brain regional specific alterations in type 2 diabetes
Source: Aging (Albany NY). 2019 Aug 26;11(16):6398–421. doi: 10.18632/aging.102196 (PMC6738403; doi:10.18632/aging.102196)
Supplement: Supplementary Figures [file aging-11-102196-s009.pdf]

SUPPLEMENTARY FIGURES

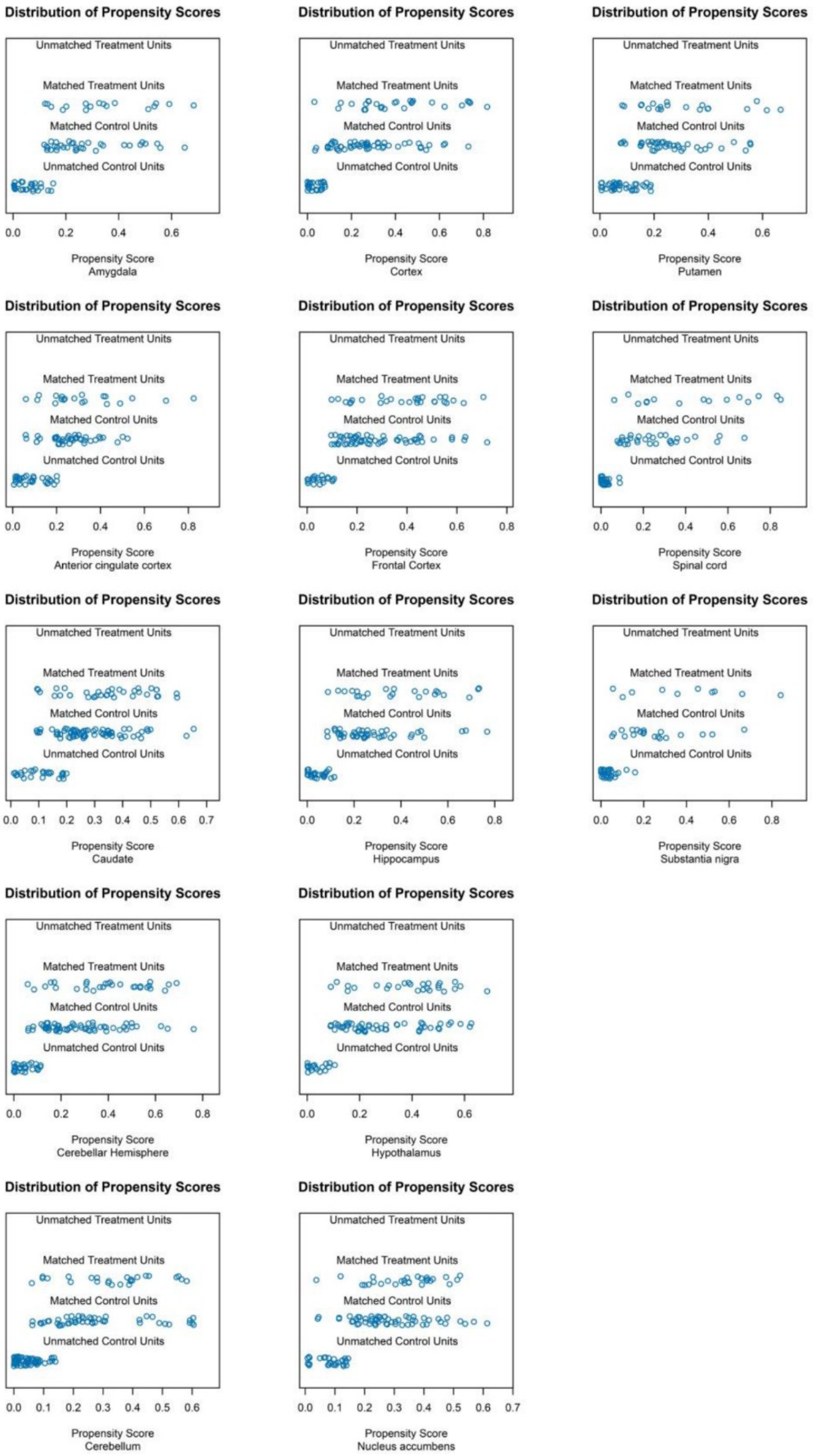

Supplementary Figure 1. Distribution of propensity scores. Jitter plot showing the distribution of propensity scores in T2D samples and matched controls for each brain regions respectively.

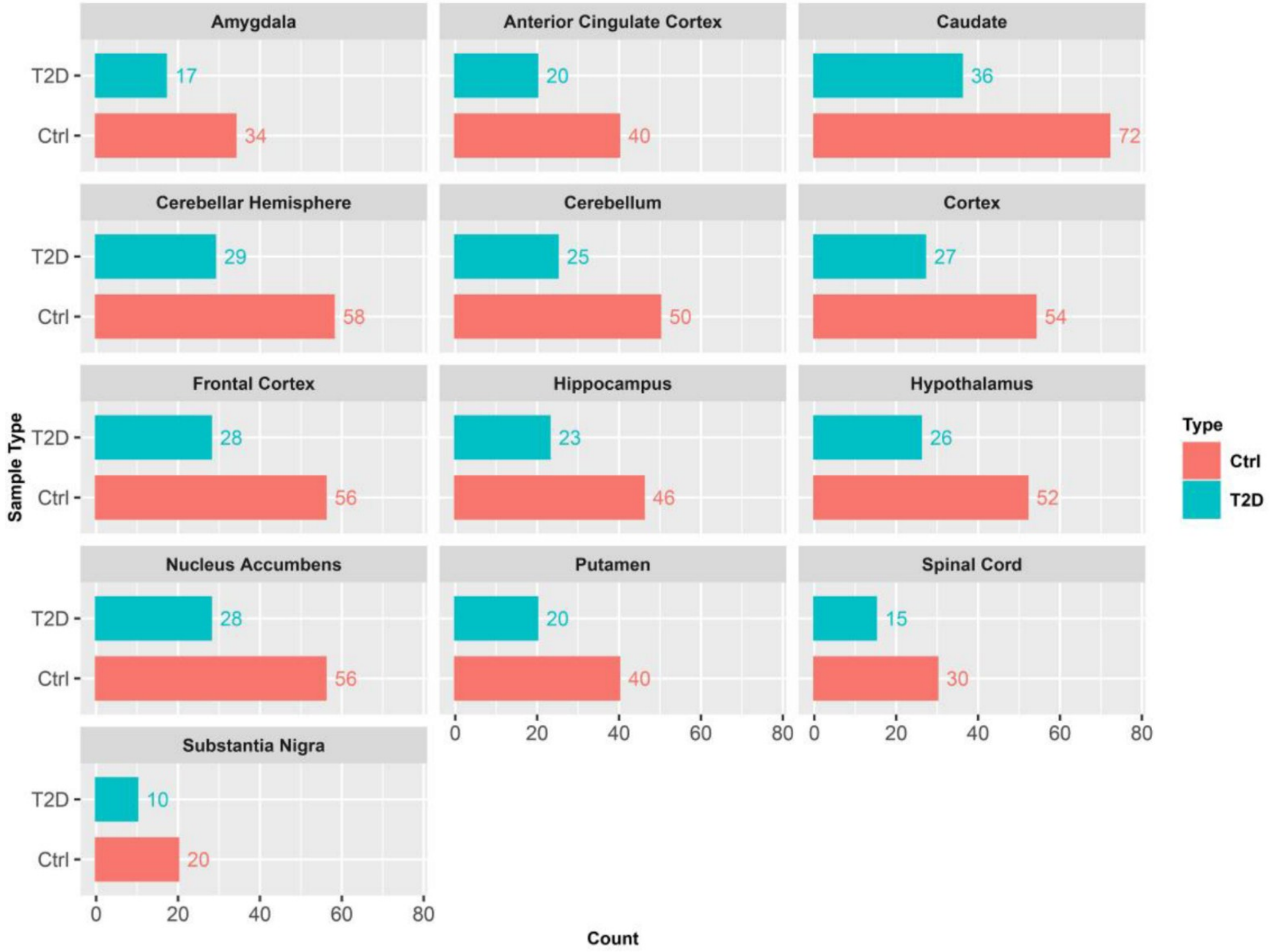

Supplementary Figure 2. Sample distribution of 13 brain regions after matching. Histograms denote the distribution of matched samples in each brain regions respectively.

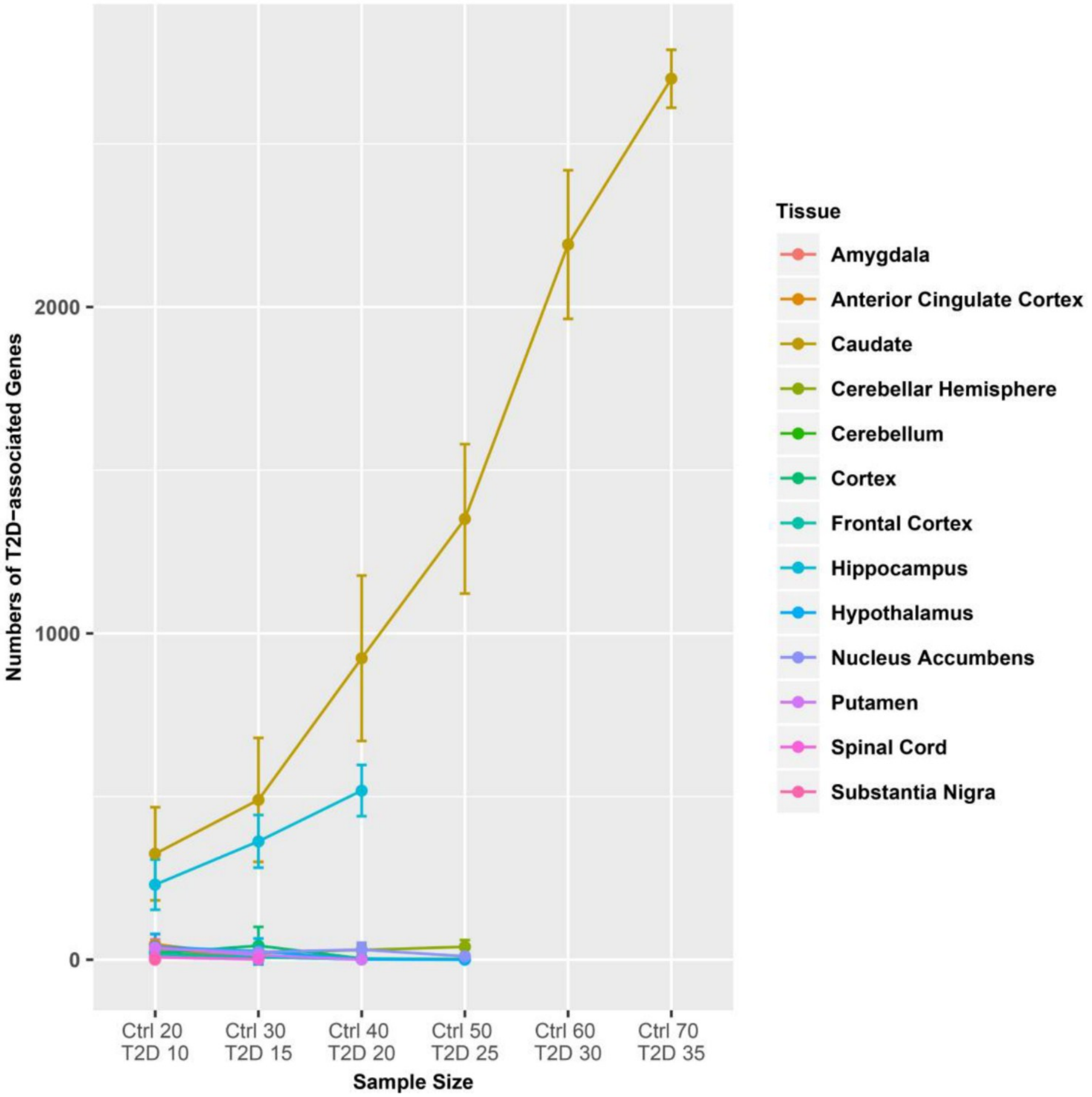

Supplementary Figure 3. Effect of sample size on detected DAGs in 13 brain regions. **The x-axis indicates the sample size of T2D and matched control groups. The y-axis indicates the average number of DAGs in 100 repetitions of bootstrapping at a 5% FDR level. The error bar indicates 95% confidence interval of group means.**

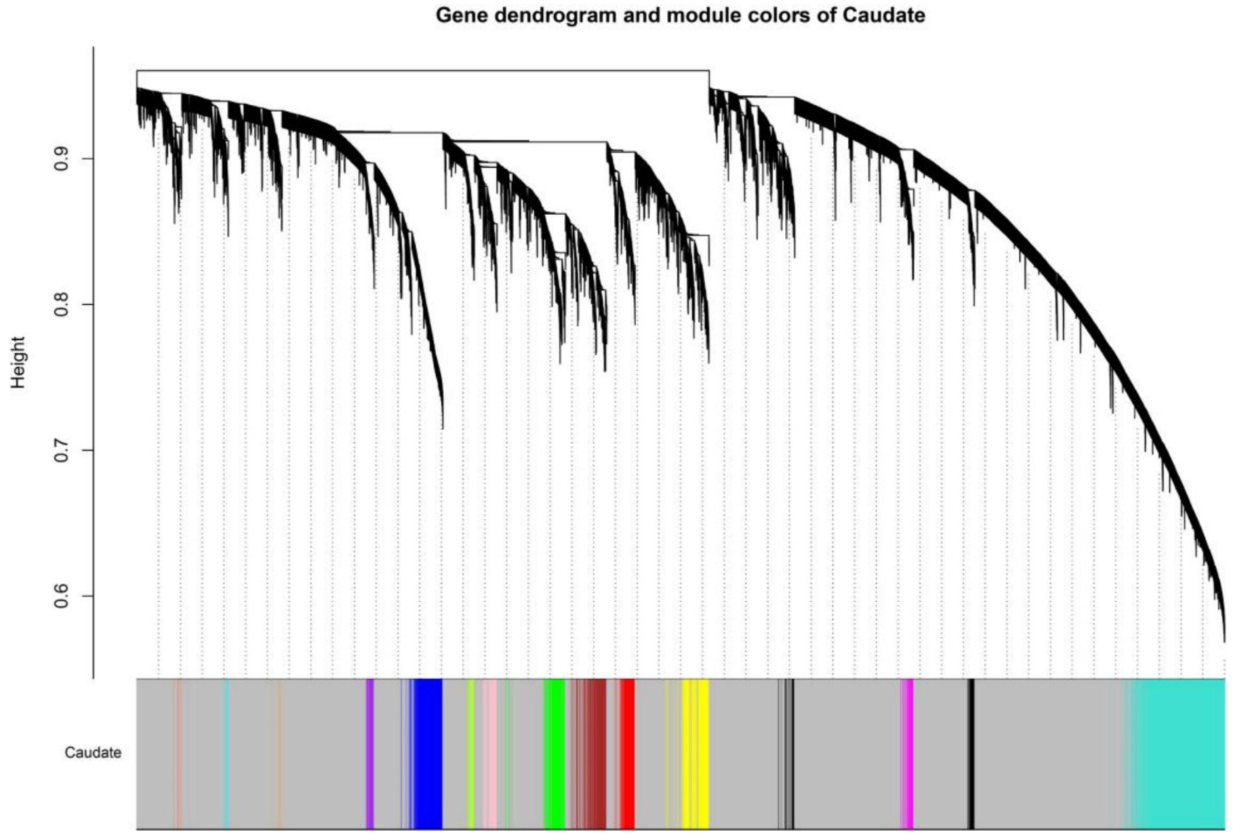

Supplementary Figure 4. Dendrogram for module assignment in caudate. The branches correspond to modules of highly interconnected groups of genes. Colors in the horizontal bar represent the modules and grey indicates unassigned genes.

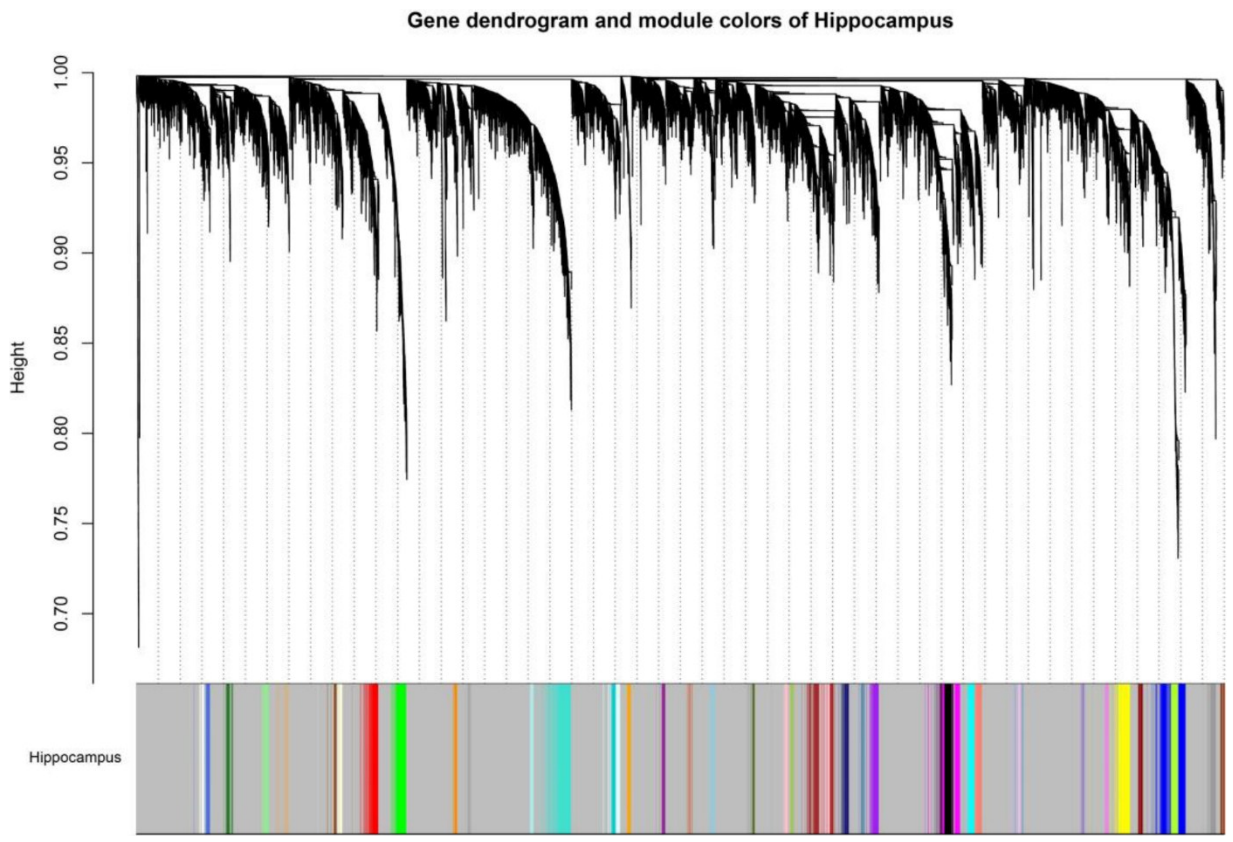

Supplementary Figure 5. Dendrogram for module assignment in hippocampus. The branches correspond to modules of highly interconnected groups of genes. Colors in the horizontal bar represent the modules and grey indicates unassigned genes.

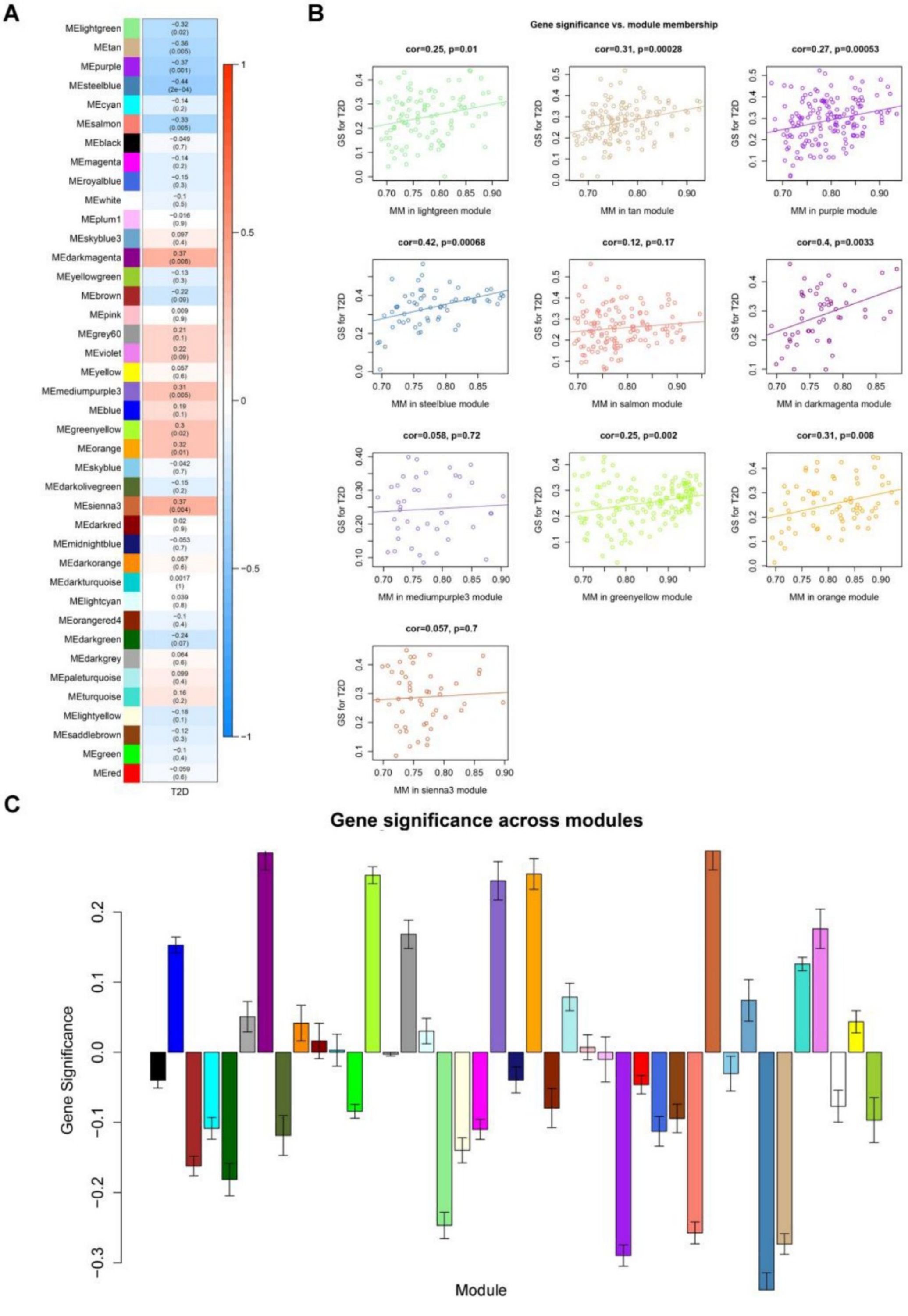

Supplementary Figure 6. Co-expression modules in hippocampus. (**A**) Each row corresponds to a module eigengene and column indicate T2D status. The table were colored by correlation according to the legend. Each cell contains the corresponding correlation and P-value. (**B**) GS vs MM plot for modules significantly correlated with T2D status. Each point corresponds to an individual gene within a given module, which was plotted by GS on the y-axis and MM on the x-axis. The regression line, correlation value and P-value were shown for each module. (**C**) Each bar indicates the average of gene significance measure for all genes in a given module.

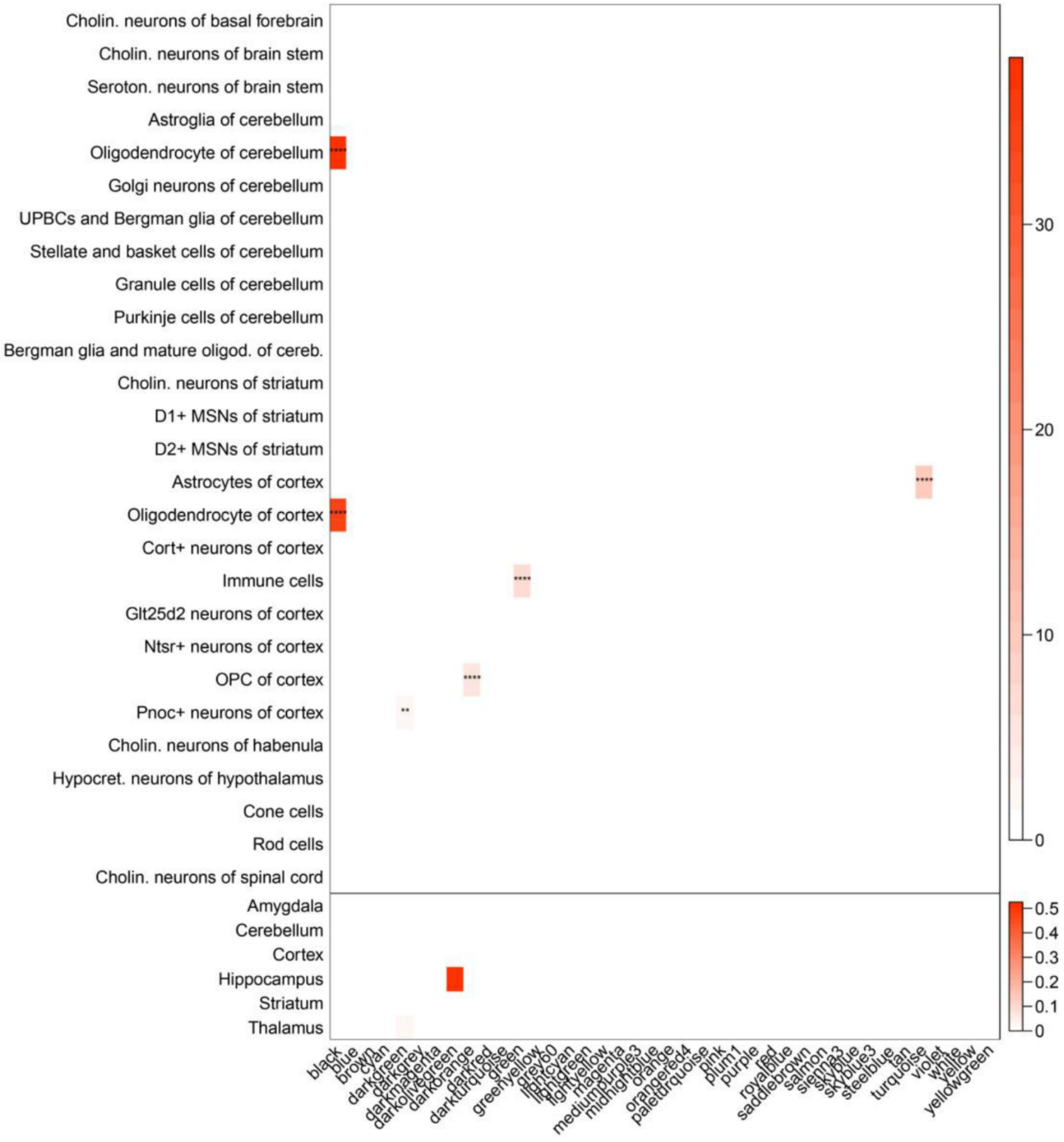

Supplementary Figure 7. Enrichment of brain regional and cell-type markers in hippocampus modules. (Top) Enrichment of hippocampus modules in markers of various neuronal and glial cell types. (Bottom) Same as above, but using markers for different brain regions. Asterisks indicate significant enrichment after Bonferroni adjustment. $^{*}P < 0.05$; $^{**}P < 0.01$; $^{***}P < 0.001$; $^{****}P < 0.0001$, Fisher's Exact test. Cholin., cholinergic; seroton., serotonergic; cereb., cerebellum; UPBCs, unipolar brush cells; oligod., oligodendrocyte; MSNs, medium spiny neurons; hypocret., hypocretinergic.
