# Supplementary material for: Comprehensive transcriptomic analysis indicates brain regional specific alterations in type 2 diabetes
Source: Aging (Albany NY). 2019 Aug 26;11(16):6398–421. doi: 10.18632/aging.102196 (PMC6738403; doi:10.18632/aging.102196)
Supplement: Supplementary Table 1 [file aging-11-102196-s002.docx]

**Supplementary Table 1. Statistics of known covariates after sample matching.**

| **Variables** | | **Amygdala** | | | **Anterior congulate cortex** | | | **Caudate** | | | **Cerebellar Hemisphere** | | | **Cerebellum** | | |
| --- | --- | --- | --- | --- | --- | --- | --- | --- | --- | --- | --- | --- | --- | --- | --- | --- |
|  |  | **Control** | **T2D** | **P-value** | **Control** | **T2D** | **P-value** | **Control** | **T2D** | **P-value** | **Control** | **T2D** | **P-value** | **Control** | **T2D** | **P-value** |
| **Sex** | **Male** | 26 | 14 |  | 27 | 13 |  | 55 | 26 |  | 41 | 19 |  | 34 | 17 |  |
|  | **Female** | 8 | 3 |  | 13 | 7 |  | 17 | 10 |  | 17 | 10 |  | 16 | 8 |  |
| **AGE** | | 61.94 | 62.18 | 0.876 | 62.4 | 62.5 | 0.960 | 60.4 | 61.47 | 0.443 | 60.79 | 62.83 | 0.120 | 61.62 | 62.12 | 0.701 |
| **Race** | **White** | 31 | 15 |  | 39 | 18 |  | 66 | 32 |  | 53 | 26 |  | 46 | 22 |  |
|  | **Black** | 3 | 2 |  | 1 | 2 |  | 6 | 4 |  | 5 | 3 |  | 4 | 3 |  |
| **BMI** | | 26.82 | 27.79 | 0.376 | 27.92 | 28.42 | 0.637 | 27.63 | 28.58 | 0.222 | 27.54 | 28.62 | 0.151 | 28.6 | 29.36 | 0.335 |
| **RIN** | | 6.86 | 6.98 | 0.461 | 6.82 | 6.81 | 0.951 | 7.72 | 7.57 | 0.285 | 7.82 | 7.86 | 0.850 | 6.94 | 6.87 | 0.685 |
|  |  |  |  |  |  |  |  |  |  |  |  |  |  |  |  |  |
| **Variables** | | **Cortex** | | | **Frontal Cortex** | | | **Hippocampus** | | | **Hypothalamus** | | | **Nucleus accumbens** | | |
|  |  | **Control** | **T2D** | **P-value** | **Control** | **T2D** | **P-value** | **Control** | **T2D** | **P-value** | **Control** | **T2D** | **P-value** | **Control** | **T2D** | **P-value** |
| **Sex** | **Male** | 38 | 19 |  | 41 | 20 |  | 33 | 14 |  | 40 | 19 |  | 40 | 21 |  |
|  | **Female** | 16 | 8 |  | 15 | 8 |  | 13 | 9 |  | 12 | 7 |  | 16 | 7 |  |
| **AGE** | | 60.85 | 62.7 | 0.170 | 60.52 | 63 | 0.045 | 61.5 | 62.48 | 0.566 | 61 | 63.65 | 0.051 | 60.48 | 61.86 | 0.358 |
| **Race** | **White** | 50 | 24 |  | 52 | 27 |  | 42 | 20 |  | 47 | 23 |  | 50 | 24 |  |
|  | **Black** | 4 | 3 |  | 4 | 1 |  | 4 | 3 |  | 5 | 3 |  | 6 | 4 |  |
| **BMI** | | 28.15 | 29.42 | 0.128 | 27.82 | 29.24 | 0.113 | 27.75 | 29.2 | 0.095 | 27.68 | 28.74 | 0.195 | 28.22 | 28.73 | 0.507 |
| **RIN** | | 6.68 | 6.83 | 0.310 | 7.36 | 7.4 | 0.807 | 6.97 | 6.92 | 0.766 | 7.1 | 7.09 | 0.978 | 7.4 | 7.38 | 0.908 |
|  |  |  |  |  |  |  |  |  |  |  |  |  |  |  |  |  |
| **Variables** | | **Putamen** | | | **Spinal cord** | | | **Substantia nigra** | | |  |  |  |  |  |  |
|  |  | **Control** | **T2D** | **P-value** | **Control** | **T2D** | **P-value** | **Control** | **T2D** | **P-value** |  |  |  |  |  |  |
| **Sex** | **Male** | 30 | 16 |  | 17 | 7 |  | 15 | 8 |  |  |  |  |  |  |  |
|  | **Female** | 10 | 4 |  | 13 | 8 |  | 5 | 2 |  |  |  |  |  |  |  |
| **AGE** | | 62.55 | 61.65 | 0.509 | 60.93 | 62.47 | 0.479 | 60.7 | 62.7 | 0.501 |  |  |  |  |  |  |
| **Race** | **White** | 36 | 16 |  | 30 | 15 |  | 16 | 9 |  |  |  |  |  |  |  |
|  | **Black** | 4 | 4 |  |  |  |  | 4 | 1 |  |  |  |  |  |  |  |
| **BMI** | | 28.07 | 28.95 | 0.357 | 27.19 | 28.32 | 0.344 | 27.46 | 28.73 | 0.325 |  |  |  |  |  |  |
| **RIN** | | 7.2 | 7.2 | 0.978 | 7.24 | 6.97 | 0.154 | 6.95 | 7.07 | 0.615 |  |  |  |  |  |  |

*Note:* The values indicate group means for AGE, BMI and RIN, while sample counts for SEX and RACE. P-value were derived from Student's t test.
