# Supplementary material for: Comprehensive transcriptomic analysis indicates brain regional specific alterations in type 2 diabetes
Source: Aging (Albany NY). 2019 Aug 26;11(16):6398–421. doi: 10.18632/aging.102196 (PMC6738403; doi:10.18632/aging.102196)
Supplement: Supplementary Tables 8 and 9 [file aging-11-102196-s005.pdf]

Supplementary Table 8. SNP-based enrichment analysis for caudate modules and sub-modules.

Module	T2D SNPs				Height SNPs			
	Size	ES	P-value	FDR	Size	ES	P-value	FDR
turquoise	1576	0.415712	0	0	1576	0.4111951	0.0497	0.13916
red	260	0.425652	0.00815	0.03325	260	0.42124715	0.2414	0.289756
magenta	156	0.444919	0.0091	0.03325	156	0.45935112	0.12445	0.2489
green	373	0.435294	0.0095	0.03325	374	0.4505773	0.04135	0.13916
tan	61	0.504785	0.01205	0.03374	61	0.46593708	0.248362	0.289756
yellow	481	0.368788	0.03835	0.089483	481	0.49281818	3.00E-04	0.0042
purple	118	0.400124	0.09525	0.1905	118	0.38965136	0.56555	0.56555
salmon	39	0.439662	0.163975	0.286956	39	0.56709814	0.069181	0.161423
greenyellow	95	0.384014	0.20735	0.306483	95	0.57951885	0.00415	0.019367
black	228	0.351542	0.24705	0.306483	228	0.42270425	0.24785	0.289756
blue	468	0.337558	0.25135	0.306483	468	0.40408793	0.2989	0.321892
brown	503	0.335818	0.2627	0.306483	503	0.47241223	0.00295	0.019367
pink	215	0.345973	0.30135	0.324531	215	0.42818555	0.22605	0.289756
cyan	37	0.315055	0.746361	0.746361	37	0.49608	0.228706	0.289756

Sub-module	T2D SNPs				Height SNPs			
	Size	ES	P-value	FDR	Size	ES	P-value	FDR
D2-MSN	71	0.485125	0.0144	0.0432	71	0.4155968	0.45245	0.49075
D1-MSN	75	0.453621	0.0355	0.04715	75	0.47593623	0.1792	0.49075
striatum	83	0.4398	0.04715	0.04715	83	0.40625075	0.49075	0.49075

Supplementary Table 9. Modules enriched in DAGs.

Up-regulated DAGs					Down-regulated DAGs				
**Module**	**GeneRatio**	**P-value**	**FDR**	**Count**	**Module**	**GeneRatio**	**P-value**	**FDR**	**Count**
blue	460/1186	0	0	460	turquoise	887/1753	0	0	887
green	57/1186	5.81E-12	1.60E-11	57	black	54/1753	6.01E-13	1.35E-12	54
purple	26/1186	5.44E-09	1.20E-08	26					

Note: GeneRatio refers to Number of intersection genes / Size of Up- or Down-regulated DAG set.

Please browse Full Text version to see the data of Supplementary Table 10.

Supplementary Table 10. Functional annotations of caudate modules (sub-modules).
